# Optimum trajectory learning in musculoskeletal systems with model predictive control and deep reinforcement learning

**DOI:** 10.1007/s00422-022-00940-x

**Published:** 2022-08-11

**Authors:** Berat Denizdurduran, Henry Markram, Marc-Oliver Gewaltig

**Affiliations:** 1 Alpine Intuition Sarl, Route de Crochy 20, 1024 Ecublens, Switzerland; 2 Blue Brain Project, École polytechnique fédérale de Lausanne (EPFL), Campus Biotech, 1202 Geneva, Switzerland

**Keywords:** Musculoskeletal simulations, Model predictive control, Deep reinforcement learning

## Abstract

From the computational point of view, musculoskeletal control is the problem of controlling high degrees of freedom and dynamic multi-body system that is driven by redundant muscle units. A critical challenge in the control perspective of skeletal joints with antagonistic muscle pairs is finding methods robust to address this ill-posed nonlinear problem. To address this computational problem, we implemented a twofold optimization and learning framework to be specialized in addressing the redundancies in the muscle control . In the first part, we used model predictive control to obtain energy efficient skeletal trajectories to mimick human movements. The second part is to use deep reinforcement learning to obtain a sequence of stimulus to be given to muscles in order to obtain the skeletal trajectories with muscle control. We observed that the desired stimulus to muscles is only efficiently constructed by integrating the state and control input in a closed-loop setting as it resembles the proprioceptive integration in the spinal cord circuits. In this work, we showed how a variety of different reference trajectories can be obtained with optimal control and how these reference trajectories are mapped to the musculoskeletal control with deep reinforcement learning. Starting from the characteristics of human arm movement to obstacle avoidance experiment, our simulation results confirm the capabilities of our optimization and learning framework for a variety of dynamic movement trajectories. In summary, the proposed framework is offering a pipeline to complement the lack of experiments to record human motion-capture data as well as study the activation range of muscles to replicate the specific trajectory of interest. Using the trajectories from optimal control as a reference signal for reinforcement learning implementation has allowed us to acquire optimum and human-like behaviour of the musculoskeletal system which provides a framework to study human movement in-silico experiments. The present framework can also allow studying upper-arm rehabilitation with assistive robots given that one can use healthy subject movement recordings as reference to work on the control architecture of assistive robotics in order to compensate behavioural deficiencies. Hence, the framework opens to possibility of replicating or complementing labour-intensive, time-consuming and costly experiments with human subjects in the field of movement studies and digital twin of rehabilitation.

**Supplementary Information:**

The online version contains supplementary material available at 10.1007/s00422-022-00940-x.

## Introduction

The ability of perceiving changes in the environment and acting accordingly with motor skills underlies the fundamental role of the central nervous system (CNS). Driven by the desire of amusement, survival or curiosity, motor skills comprise the range of all possible actions that can be executed to achieve any of the goals set by oneself. A significant feature of (CNS) is that a new motor skill can be added on top of the existing ones, by which it re-organizes the sequence of learned actions to enrich the capabilities of motor control (Hikosaka et al. 2002). These learned motor skills can be possessed and last throughout the entire lifetime (Park et al. 2013; Romano et al. 2010) which indicates that (CNS) has the ability to store long-term motor skills based on a long-term retained plasticity mechanism (Dayan and Cohen 2011; Ungerleider et al. 2002). Yet, the division of labour in acquiring new motor skills or execution of already integrated ones and the details of the role of perception/sensory integration among all distributed motor areas are still the interest of active research (Kawai et al. 2015).

Computational models of human motor control systems aim at progressing towards understanding and revealing the underlying structure of this scientific challenge; human mobility and its capabilities as well as its limitations. This scientific challenge has two layers, one being the neuroscientific perspective, second the biomechanical one due to its unique antagonistic muscle actuation at the skeletal system. Thus, computational models of motor control systems have been proposed by using neuromechanical simulations where the aim has been to evaluate and validate physically plausible movements of a human musculoskeletal system (Fregly et al. 2007; Steele et al. 2015; Priamikov et al. 2016).

Musculoskeletal simulations usually focus on one region of interest such as lower or upper extremities. Models of movements of the lower extremities typically focus on periodic movements such as the emergence and transition between different gaits as well as jumping, hopping and running (Pandy et al. 1990; Geyer et al. 2003; Geyer and Herr 2010; Lim et al. 2003; Ackermann and Schiehlen 2006). Models for the upper extremity of the human body are more versatile. Some focus on kinematic studies of the human arm and its control of motion coordination (Seth et al. 2003; Garner and Pandy 2001), and some consider the contribution of the rather complex shoulder anatomy on arm movements (Vander Helm 1994). Other upper extremity research is dedicated to hands, arms and finger movements (Rosenbaum et al. 2001; Santos and Valero-Cuevas 2006; Friedman and Flash 2009). Apart from dynamics and kinematics of the movements of human extremities, the control scheme of the neural motor control system is also a central interest of musculoskeletal studies (Vander Helm et al. 2002). Based on these studies, one of the lately emerging fields is the transformative studies where next generation prosthesis and orthopaedic solutions are provided (Cavallaro et al. 2005).

The use of reinforcement learning (RL) for musculoskeletal control has been gaining attention among several studies (Jaśkowski et al. 2018) along with metaheuristic optimization methods (Lee et al. 2014). The problem of scalability of meta-heuristic optimization methods, such as stochastic optimization methods, limits the possibility of studying complex dynamic models. Instead, deep reinforcement learning (Deep RL) implementations are providing more promising results. A common strategy in Deep RL studies is to obtain a solution that maximizes the cumulative reward regardless of the movement. Most training schemes for musculoskeletal models use reference motion learning, similar to the motor learning by imitation, where the reference motions are created by humans or recorded with motion capture techniques, others use hand crafted motion captured data such as in Geijtenbeek et al. (2013); Coros et al. (2011). Recently, with the integration of RL into neural networks(NN) with more than two hidden layers (Mnih et al. 2015) (a.k.a Deep RL), there has been significant improvements and successful solutions for the control of high-degree articulated robots and musculoskeletal systems (Peng and vande 2017; Peng et al. 2017, 2018; Lee et al. 2019). It has been shown that these models have the capability of reproducing highly complex human behaviours, such as walking, flipping, reaching and grasping. The performance of these models highly depends on the construction of the control architecture, for instance (Peng and vande 2017) showed that different mechanisms to control the actuators significantly alter the success of these simulations such that muscle and proportional-derivative controller succeeds over torque control. However, due to excessive number of parameters in muscle control, the policy learning of these musculoskeletal systems with Deep RL methods is more challenging and it requires longer training procedures than torque control architecture. It also has been reported that a promising policy learning for a musculoskeletal running is achieved with a similar learning architecture (Kidziński et al. 2018), however in 2D dynamics.

Following the development of the Deep RL methods, revolutionary advances have occurred in musculoskeletal learning, where higher degrees of freedom has been extensively started to be considered successful. Unlike the previous problem definitions (Lee and Terzopoulos 2006; Sok et al 2007; Yin et al. 2007), Deep RL methods are not only applied to musculoskeletal control problems but they have also demonstrated great success in torque controlled dexterous robots, including walking and running problems (Peng et al. 2017), as well as control of different morphologies (Won et al. 2017). Lately, the imitation RL has also been extensively used in musculoskeletal studies where user defined key-frame motions are used to generate the synthetic data one by one (Geijtenbeek et al. 2013; Coros et al. 2011; Peng and vande 2017). Apart from plain implementation of Deep RL methods, there also has been an attention to develop hierarchical control architectures to study sparse reward problems (Levy et al. 2018). Despite the great success of Deep RL methods in musculoskeletal simulations with high degree-of-freedom and high number of muscles, Deep RL methods still don’t scale up to the level of human motor control with excessive degree-of-freedom and abundance muscles. The concern about these methods rise due to the fact that Deep RL methods are not sample efficient, that is, they are too slow to match up with exponential growth of the complexity of the human musculoskeletal simulations. Notwithstanding the caveats, recent improvements in Deep RL, namely Episodic Deep RL, (Botvinick et al. 2019; Gershman and Daw 2017; Pritzel et al. 2017) and Meta-RL (Andrychowicz et al. 2016; Finn et al. 2017), encourage higher complexity of the musculoskeletal simulations (Lee et al. 2019).

In this work, we incorporate the theory of optimal control into optimization procedure of musculoskeletal movement control where we map desired joint angles to muscle activations with Deep RL.. The principle idea of the integration of optimal control theory is to address one of the well-known problems of biomechanical control, known as degrees of freedom or motor equivalence problem (Bernstein 1966). The problem indicates that there isn’t a one-to-one correspondence between a desired movement and a kinematic solution to that problem. CNS chooses a solution among abundant combination of muscle recruitments and joint coordination. Notwithstanding, CNS continuously adapts its solutions to changing conditions given that the body is under constant development and ageing. The motor equivalence problem stems from the fact that there are redundancies in almost all parts of the motor control system, such as each joint is controlled by multiple antagonistic muscles. Besides the redundancy of the muscle control, there are kinematic redundancies as well, such that a movement can be obtained with different joint trajectories, velocities and accelerations. Based on this approach, there have been several hypotheses suggested among them are muscle synergies, equilibrium point and threshold control, force control and internal models, uncontrolled manifold and last but not least optimal control theory (Ting and McKay 2007; Feldman 1966, 1986; Asatryan 1965; Ostry and Feldman 2003; Scholz and Schöner 1999).

The hypothesis of optimal control theory (OCT) relates the solution of motor equivalence to the objective of minimizing a certain cost in a principled way. The optimal control hypothesis aims at giving scrupulous explanations to the details of motor control system such that how CNS deals with redundancy, uncertainty and link between invariance and task performance (Guigon et al. 2007; Körding and Wolpert 2004; Harris and Wolpert 1998; Nitschke et al. 2020; Van DenBogert et al. 2011). The distinctive feature of OCT from other hypotheses is that the explanation of motor behaviour is connected not only to evolution but also motor learning by the definition of objective functions (a.k.a task performance, cost, cost-to-go functions). There are also studies indicating neural control circuits inherently learn the kinematic as well as dynamic properties of the musculoskeletal system, as these are highly variable throughout the life of an animal (Todorov and Jordan 2002).

**Fig. 1:**
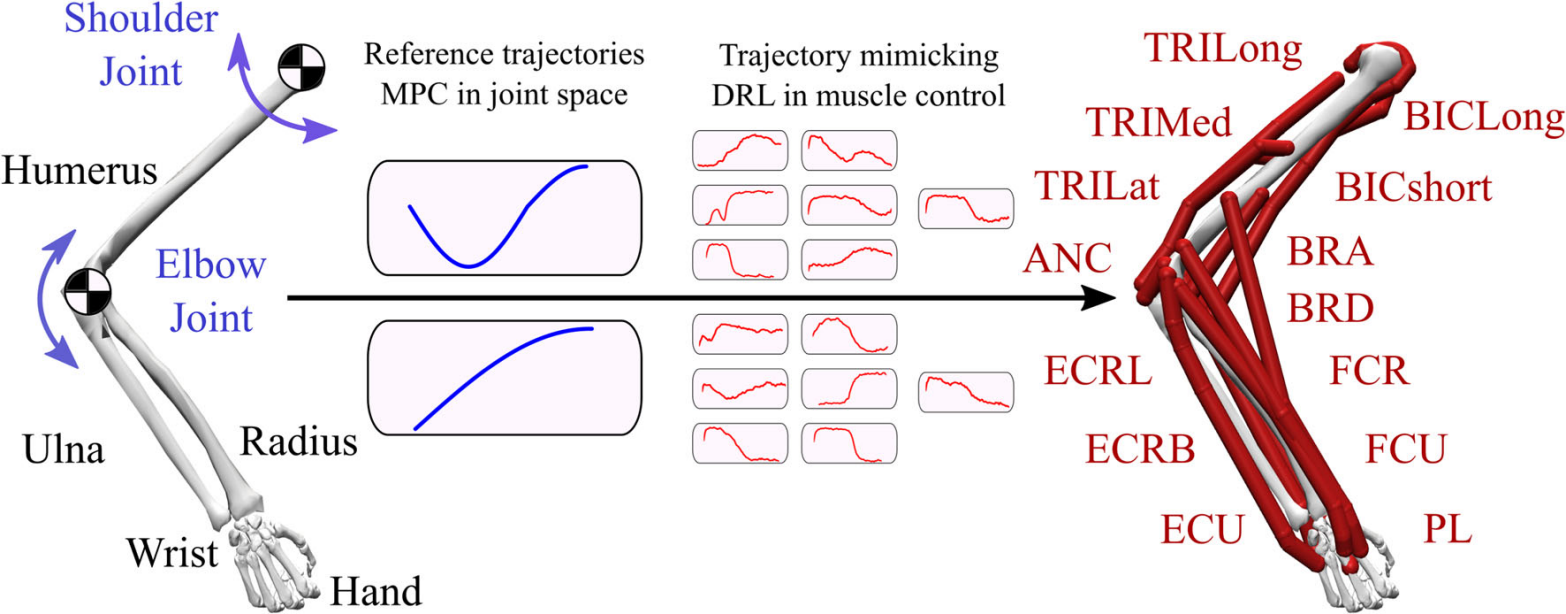
Musculoskeletal human arm model. Skeletal representation of the arm with the bones of humerus, ulna, radius, wrist and hand on the left. Shoulder and elbow joints are in blue. Model predictive control is applied to skeletal system to obtain reference trajectories. These joint trajectories are used as supervised signals in trajectory mimicking problem definition of deep reinforcement learning to obtain time-dependent muscle activities. Shoulder and elbow joints are controlled by 14 extensor and flexor antagonistic muscles on the right; TRILong, TRIMed, TRILat, BICLong, BICShort, BRA, ANC, BRD, ECRL, ECRB, ECU, FCR, FCU, PL. Simulation and visualization of the musculoskeletal system are done with OpenSim 4.0 (Seth et al. 2018)

The principal contribution of this work is to introduce a new optimization and learning framework for studying neural control of movement. Trajectories that are found by OCT can give us the information about joint movements at the torque level, whereas the first goal of the reverse engineering the motor circuit is to find out what are the force generations with redundant muscles. This in fact can be regarded as an ill-posed problem, where the objective is to identify the multiple driving forces coming through redundant extensor and flexor muscles which yields a single dimensional behaviour. In order to examine the optimality of the movement trajectories, we focus on the OCT to find out the movement trajectories in the level of joints. We use these signals as reward function for RL to find out the level of stimulus given to extensor and flexor muscles which is in charge of controlling skeletal joints. Solution of this ill-posed problem yields the joint control with multiple extensor and flexor muscles. Our approach not only allows us to generate necessary reference trajectories with optimal control but also solve redundancy of the musculoskeletal systems. The objective of utilization of the RL in this study is to find time dependent stimulus for muscle contractions given a desired trajectory of the musculoskeletal system. Therefore, the principle idea behind the proposed learning and optimization framework can be summarized as to utilize optimum joint trajectories as supervised signal and use them to obtain a policy function for muscle control. These reference motions can be either human and animal motion data or synthetically generated motions such as a solution of an optimal control formulation. We show that integration of state-of-the art RL methods with OCT that are linked through reward functions is capable of addressing this inverse imitation learning problems.

## Methods

In this section, we describe the details of the musculoskeletal arm model, the nonlinear model predictive control (MPC) approach to obtain the solution of torque control with skeletal dynamics and how we integrate this solution into Deep RL in order to achieve musculoskeletal control in muscle space.

### Human musculoskeletal arm model

The musculoskeletal model of the human arm has been simulated with an open source software, called OpenSim (Seth et al. 2018) and the model that we used in this research is an adapted version of the upper extremity OpenSim model (Holzbaur et al. 2005). The adapted version of this model has 2 degrees-of-freedom (DOF), controlled by 14 muscle-tendon units (MTU)s in the shoulder elevation and elbow flexion, see Fig. 1. In our adaptation, the coupling constraint of the shoulder joint is adjusted to obtain stable rotation. In addition, remaining rotational joints in the shoulder and all rotational joints in the hand are locked due to our interest in 2D movement of the shoulder and elbow joints. We simulate the model in two different settings; skeletal control with actuators attached to the joints in MPC simulations (Fig. 1A) and the musculoskeletal control with 14 MTUs in Deep RL procedure (Fig. 1B).

**Fig. 2:**
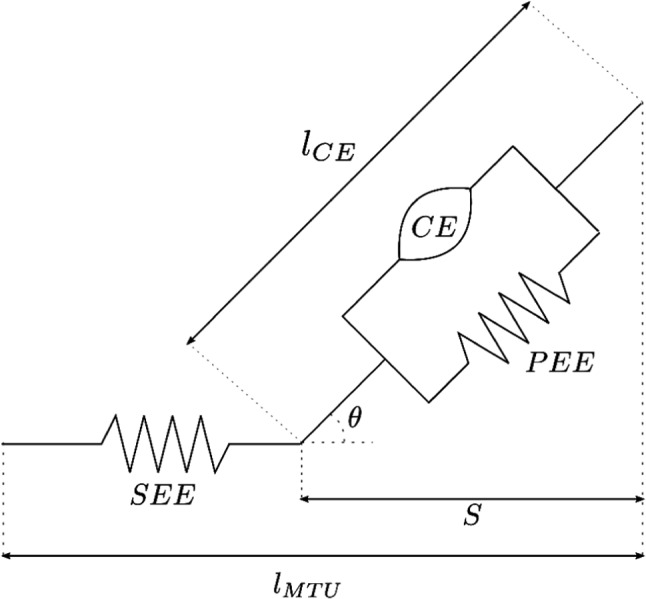
Muscle-tendon unit. The MTU is comprised of muscle and tendon unit with a length described by ($l_{MTU}$). In muscle unit, there exists a contractile element (CE) and passive elastic element (PEE), together with a length of ($l_{CE}$). Tendon is connected to muscle unit as series elastic element (SEE). The pennation angle and state variable of contraction dynamics are $\theta$ and *s*, respectively

In our musculoskeletal simulations, the dynamics of the MTU is described by a Hill-type muscle model of which all the parameters are taken from Holzbaur et al. (2005). We refer readers to supplementary material (Sect. 2) for a detailed description of the OpenSim model and muscle parameters of the upper extremity model. The MTU defines the dynamic properties of the muscle that is modelled as a serially connected combination of an elastic tendon (SE) and a muscle unit. The SE creates tendon force and it takes part in the force-length and force-velocity profile of the muscle. The muscle unit is again composed of two parts: a contractile element (CE) which represents the dynamics of the activation and corresponding contraction along with a parallel elastic element (PE) which becomes active in case of excessive stretches in the contractile element (see Fig. 2).

### Model predictive control

One of the goals of our proposed optimization and learning framework is to use desired movements as supervision signals for a computational motor control problem. In this framework, either a kinematic information of a human subject or a synthetic movement data can be used as a source signal. In case of lacking the kinematic data found by human motion capture experiments, an optimum trajectory found by MPC can give us the information about joint movements at the torque level, hence we integrated the MPC to find out the movement trajectories in the level of joints to be used as reward signal in the deep reinforcement learning implementation.

**Fig. 3:**
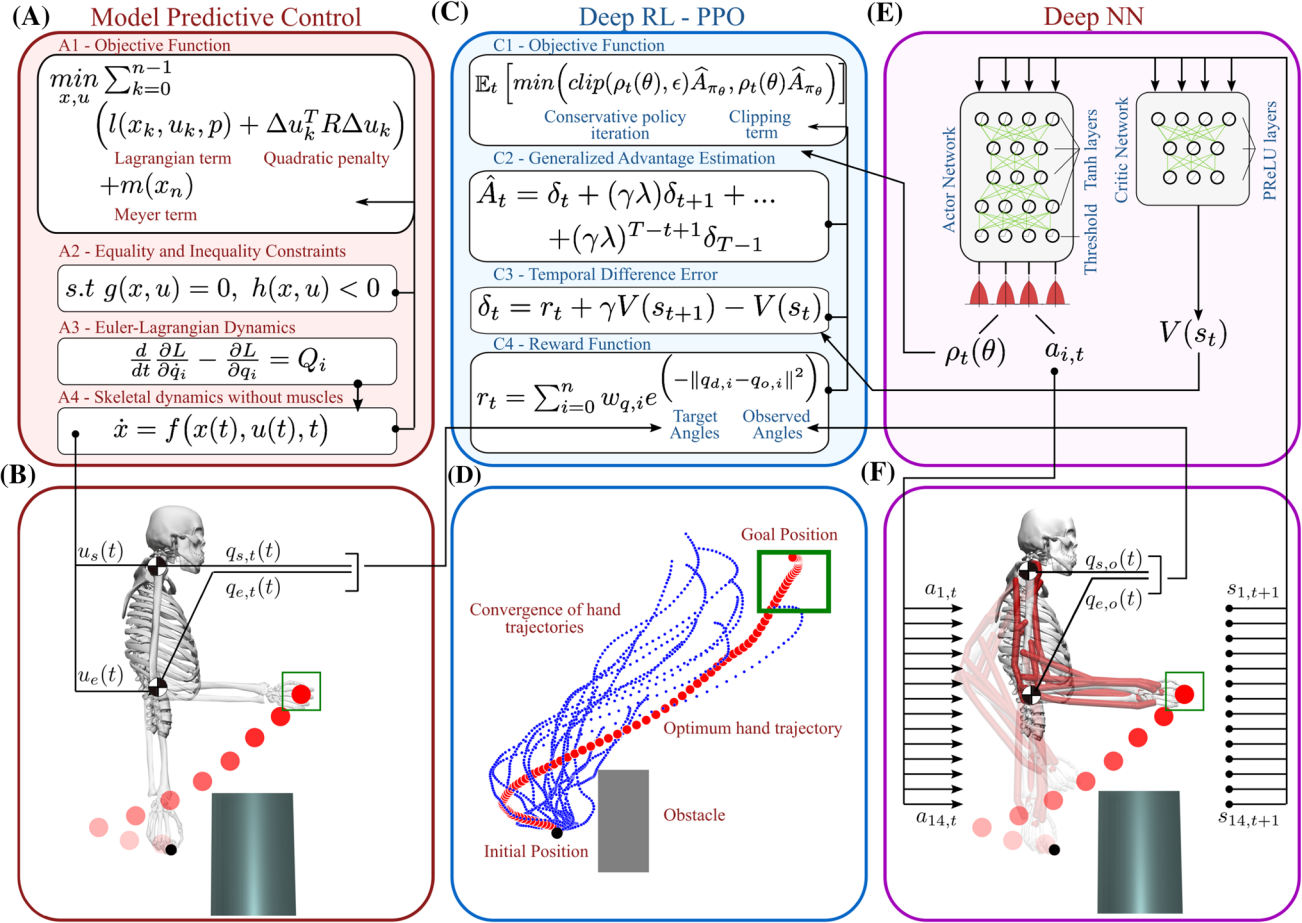
Schematic of the optimization and learning framework for musculoskeletal control problems. **A** Flow of equations for the MPC in skeletal systems. We start the solution of the MPC by writing the Euler-Lagrangian Dynamics (A3) of the skeletal system without muscles. Solving Euler-Lagrangian equation, one can the obtain the skeletal dynamics without muscle control (A4) that constraints the solution of the MPC (A2). This system dynamics then incorporated in the objective function of MPC along with user defined equality and inequality constraints (A2). **B** Solution of the MPC yields the joint trajectories and velocities with necessary torque activities that provides the supervised signal to reward function of the Deep RL. **C** A variant of policy gradient methods, PPO is written as a minimization of target angles provided by MPC and observed angles from musculoskeletal system. **D** The movement of the hand gradually converges to optimal hand trajectory after 200 batches. **E** An actor-critic architecture of a deep neural network is used to integrate the solution of PPO. Whereas the critic network evaluates the solution by assigning a value to each decision, actor network controls 14 antagonistic muscles within a closed-loop architecture. F. Both actor and critic networks receive the states of the muscles as input and actor network activates the muscles to generate the desired movement in musculoskeletal system

The control problem of the skeletal system with torque actuators is governed by a nonlinear dynamical system which is defined by a continuous differential equation:

1 $$\begin{aligned} {\dot{x}}=f\big (x(t),u(t),t, p_{f} \big ) \end{aligned}$$

where *t* represents time, parameters are denoted by $p_f$, $x(t) \in \Re ^n$ describes the state vector, $u(t) \in \Re ^m$ denotes the control input and the derivative of the state vector is given by ${\dot{x}}(t) \in \Re ^n$ and *f* is a Lipschitz continuous vector field. In this study, we assume that the vector field *f* is approximated by a set of difference equations with small time interval $\varDelta t$ using Euler method:

2 $$\begin{aligned} x_{k+1}=x_k + dt {\hat{f}}(x_k,u_k,p_{fk}) \end{aligned}$$

with ${\hat{f}}$ describes the approximated dynamical system. The goal of the MPC is to obtain a state and input trajectory, *x*(*t*), *u*(*t*), that a user defined objective function, *L*(*x*(*t*), *u*(*t*)) is minimized while respecting the state dynamics, equality and inequality constraints (see Fig. 3A). The objective function is described by a quadratic cost over a fixed time horizon and it is shifted by one time step until the end of the duration of the simulated movement:

3 $$\begin{aligned}&\underset{x,u}{min}\sum _{k=0}^{n-1}\Big (l(x_{k},u_{k},p_{l})\nonumber \\&\quad +\varDelta u_{k}^T R \varDelta u_{k}\Big )+m(x_n) \end{aligned}$$

where $l(x_{k},u_{k},p_{l})$ is the Lagrangian term that describes the minimization along the duration of the horizon, $\varDelta u_{k}^T R_l \varDelta u_{k}$ describes the quadratic penalty on the control inputs and $m(x_n)$ represents the Meyer term to define the final point objective. Minimization of the cost function is subject to following equality and inequality constraints on lower and upper bounds on states and inputs:

4 $$\begin{aligned}&x_L(t) \le x(t) \le x_U(t) \nonumber \\&u_L(t) \le u(t) \le u_U(t) \end{aligned}$$

while considering the system dynamics, given in Eq. 1. Figure 3A shows that the objective function, Eq. 3, takes the equality and inequality constraints, Eq. 4, into consideration while restricting the directions of the solution within the feasible range of skeletal dynamics, Eq. 1. Moreover, final point goals, such as endpoint position control of the skeletal arm, will be solved by the Meyer term in Eq. 3 as it is evaluated outside the summation of the Lagrangian term and quadratic penalty on control inputs. We also weight the Lagrangian term and quadratic penalty with predefined coefficients, $(p_{l}, R_{l})$, and consider them in the set of hyperparameters to be defined in each experiment. Control inputs, a set of torque values for each joint, as well as the trajectory of the all joint angles are obtained by minimizing Eq. 3 using an MPC package, called do-mpc (Lucia et al. 2017). The joint angles that are obtained by MPC in each timestep (Fig. 3B) then used as reference trajectories and considered being a minimization problem in the formulation of the Deep RL which can be followed in Fig. 3B. We used the implementation of direct collocation method from do-mpc (Lucia et al. 2017) package to solve the nonlinear programming that defines the model predictive control formulation. We only adjusted this implementation for each of the problem settings that we are interested in. An example implementation of the above equations for the skeletal control problem can be found in Sect. 3 of the Supplementary material.

### Deep reinforcement learning

Recently, policy gradient methods have been widely used RL methods in continuous-time and space control problems (VanHasselt and Wiering 2007). One of the practical advantages of the policy gradient is to obtain the policy function without requiring a value function to select the appropriate action at a given state, although a value function can still be used to learn the policy parameters but not necessarily used in action selection. Besides practical reasons, with policy gradient methods, approximated policy function can yield a deterministic policy, as well as action selection can be obtained with arbitrary probabilities which proved to be necessary for problems that require stochastic policy. One of the most important theoretical advantages of the policy gradient methods is to avoid catastrophic change in action probabilities by allowing the change of parametrized policy function to be adjusted smoothly. Hence, we used Deep RL to map the joint space actions into muscle space actions to transfer the joint trajectories into musculoskeletal system albeit with muscle actuations.

The main idea behind policy gradient methods is to obtain a parametrized policy function $\pi _{\theta }(\tau )$ that is parametrized by a vector $\theta$, with applying stochastic gradient ascent on cumulative reward with respect to this parameter vector $\theta$ that can be expressed as:

5 $$\begin{aligned} \underset{\theta }{max}\mathop {{\mathbb {E}}}_{\tau \sim \pi _{\theta }}[r(\tau )] \end{aligned}$$

where each rollout is represented by a sample from the policy function distribution over a trajectory $\tau \sim \pi _{\theta }$. Hence, the gradient that we need to calculate for the optimum policy function can be obtained by applying log likelihood trick.

### Deep neural network and implementation details

The state space of the RL implementation was defined by the length and velocity of the contractile elements in muscle model ${\mathcal {S}}=[l_{CE,1},...,l_{CE,N}, {\dot{l}}_{CE,1},...,{\dot{l}}_{CE,N})$. The output of the neural network ${\mathcal {A}}=[a_{1},...,a_{N}]$ becomes the stimulus vector given to the activation dynamics of the hill-type muscle modes which is a first-order differential equation which incorporates the neural delay (see Supplementary material 2). The transition probability is expressed by the probabilistic policy function ${\mathcal {T}}=\pi _{\theta }(\tau )$ with a continuous reward function. There exists several approaches to write a reward function for RL problems. It can be defined as a high-level goal such as obtaining a forward motion or reaching a specific position in joint space. It can also be engineered and defined as a combination of several objectives, such that a minimization of energy while obtaining a goal position. In our approach, which is similar to imitation learning problems, we defined the reward as a measure of how close the state of musculoskeletal system to a given joint trajectory obtained by MPC. Therefore policy search is not defined as a high-level goal, e.g. position specified reward function, instead a desired trajectory is given as a reward function for RL formulation to be imitated. Consequently, reward function is the sequence of joint positions and velocities of the musculoskeletal system defined as a minimization of the difference between the given and actual trajectories. Defining the reward function as a minimization of motion trajectories and the actual trajectories corresponds to an instance of an inverse RL formulation. Finding out complete reward function that would yield a solution of the inverse RL problem, however, is beyond the objective of this study. It is also known that the characteristic of human arm movement is creating a smooth trajectory with minimal trembles (Flash and Hogan 1985; Shadmehr et al. 2005). To enforce the solution of Deep RL to generate this smoothness in the movement, we also integrated a term that penalizes high acceleration terms into our reward function formulation as a regularization term to minimize the trembling movement and enforce smooth movements. After this intuitive description, reward function can be defined as a sum of weighted differences between desired and actual trajectories not only for the differences between positions but also the velocities:

6 $$\begin{aligned} r_t&= \sum _{i=0}^{N} w_{q,i} \Big (-\left\| q_{d,i}-q_{o,i} \right\| ^2 \Big ) \nonumber \\&\quad + \sum _{i=0}^{N} w_{{\dot{q}},i} \Big (-\left\| {\dot{q}}_{d,i}-{\dot{q}}_{o,i} \right\| ^2 \Big )\nonumber \\&\quad + \sum _{i=0}^{N}w_{\ddot{q},i} (\ddot{q}_{o,i})^2 \end{aligned}$$

where $q, {\dot{q}}, \ddot{q}$ denote the positions, velocities and accelerations of the joints, respectively, $w_{q,i}, w_{{\dot{q}},i}, w_{\ddot{q},i}$ are the weights of each minimization objective, and also *N* represents the total number of steps during a given simulation to complete a movement from the initial position to the end position. L2-norm is used in order to always obtain a scalar value for the error between desired and actual trajectories. Initial state of the majority of the experiments are $s_0=(0,0)$, otherwise it is stated at the corresponding explanation of the experiment.

The deep neural network in this study is relied on an actor-critic architecture. It is based on the implementation of Engstrom et al. (2019) with necessary extensions to adjust the architecture for musculoskeletal control problems. We used feedforward layers for both actor and critic networks with five and three hidden layers, respectively. The neurons in the layers of critic network are activated by PReLu (He et al. 2015) with a linear layer that receives the output of the network and transmits a single value that represents the value function $V(s_t)$. Similar design principle is applied to actor network; however, the neurons in the hidden layers are activated by Tanh function. Most important detail in the actor network is the last layer which provides the probabilistic distribution of the action values $\rho _t(\theta )$. Here we deployed a threshold function to be able to scale the values of the distribution between [0,1] to match the activation range of hill-type muscle models. Details of the PPO implementation are given in Sect. 3 of the Supplementary material.

To show the capabilities of the proposed learning and optimization framework, we addressed four different scenarios with an increasing complexity, starting from investigating the invariant properties of human arm movements, precise timing control, weight lifting to the obstacle avoidance.

**Fig. 4:**
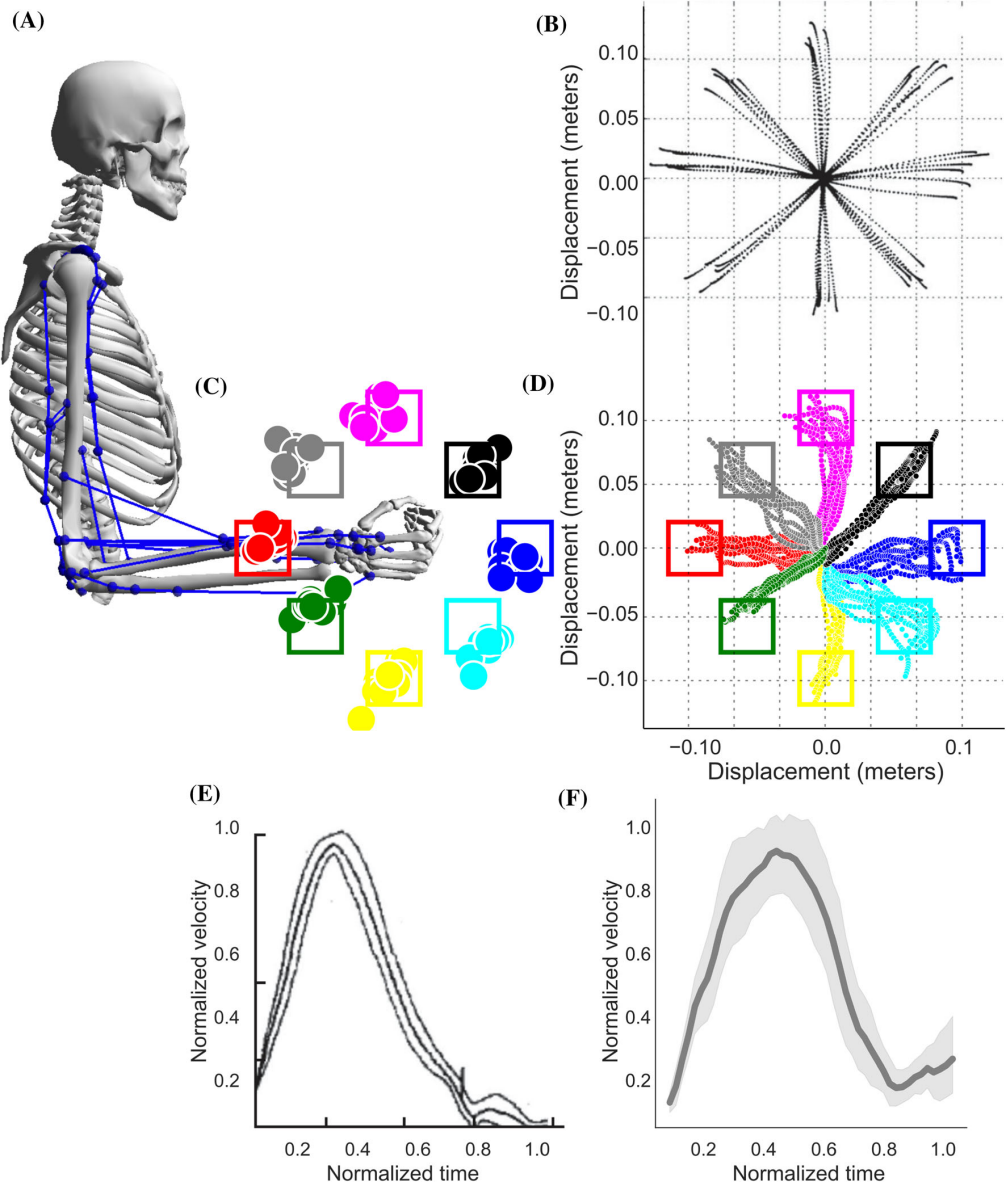
Eight equidistant centre-out reaching experiment. **A** The schematic representation of the human musculoskeletal arm, movement in the sagittal direction. **B** Human data is taken and adapted from Shadmehr et al. (2005) “Copyright 2005 Society for Neuroscience”. **D** Simulation results of eight reaching experiments, trained neural network is executed eight times to analyse the variability in the model. Each reaching target is indicated with different colours. **D** With same colour code as in **C**, however only the end points of the hand trajectory are given on top of the musculoskeletal schema. **E** Normalized velocity profile of tipping point of the hand in human data, taken from Shadmehr et al. (2005) “Copyright 2005 Society for Neuroscience”. **F** Simulation results of the velocity profile of the tipping point of the hand. The mean and variance of the simulation results show strong correlation with experimental results

## Results

In this study, based on the optimal control hypothesis and deep reinforcement learning, a broad range of movement generation with a musculoskeletal system is examined. We perform several experiments of varying complexity to assess the quality of our learning and optimization framework that we introduced in Sect. 2.

### Invariants of movement in human arm

In order to compare the ability of our learning and optimization framework, we investigated the characteristic properties of human arm movement (Shadmehr and Mussa-Ivaldi 1994; Todorov and Jordan 2002; Morasso 1981), known as invariant of movements of human arm. In Fig. 4, we compared the experimental results and the ability of our learning and optimization framework in replicating these findings. The task that we performed in this experimental setting is called centre-out reaching task with eight equidistant targets (see Fig. 4A and C). The goal of the musculoskeletal arm movement is to mimic the trajectories that have been observed in human arm movement (Shadmehr et al. 2005) and reach eight different end positions that are indicated with different colours.

Although it is possible to encode human motion capture data, we focused on the cost function of Markov decision process (MDP) in Eq. 3 and write the cost functions to obtain supervised signals that are the solutions of the MDP, which in turn optimum solutions if the solution satisfies the minimum value of the objective function while satisfying equality and inequality constraints for each centre-out targets. From now on, we call these trajectories as optimum supervised signals since we obtained the minimum value of the each objective while satisfying the above-mentioned constraints. In this experiments, the cost function is written as follows;

7 $$\begin{aligned} \underset{x,u}{min}\sum _{k=0}^{n-1}\Big ( (x-x^{*})^2 + \varDelta u_{k}^T R \varDelta u_{k} \Big ) + (x-x^{*})^2 \end{aligned}$$

where one can use the same expression for the Lagrangian and Meyer term, $(x-x^{*})^2$, in this expression $x, x^{*}$ denotes the current joint positions and target joint positions, respectively, $\varDelta u_{k}$ is the change of input value between two consecutive inputs and R is a diagonal matrix where the diagonal values are chosen “0.1”. The Meyer term is also chosen as the difference between the current joint positions and target joint positions, which has to be minimum at the last step of the timestep.

Afterwards, RL agent used these supervised signals to obtain muscle activities to replicate movement with muscle coordination. Figure 4B and D shows the displacement of the human hand tip point and simulation results of the musculoskeletal arm movement in 2D space. For each reaching target, the learned model is executed ten times to observe the variability of the movement. This variability is the result of the stochastic neural network that has been trained with proximal policy optimization. Even the policy function is stochastic, the neural network controls the arm movement within a standard deviation that is reported in each experiments. While the reaching end points are given in Fig. 4C, entire trajectories of each movement can be observed in Fig. 4D. The success rate of reaching goal position is measured with a $\%10$ of variability as indicated with coloured squares in Fig. 4C. The model performed approximately $\%89$ of success rate for reaching the target position while the correlation ratio between observed data and model performance is 0.91 across the mean and 0.78 with respect to variance. It also has been shown that the movement of human arm has an inverse bell-shaped velocity profile and it consistently appears in distinctive joint position profiles (Morasso 1981), as it is indicated in the experimental data given in Fig. 4E. We also observed similar velocity profile of the learned model as it can be seen in Fig. 4F. The velocity profile of the eight equidistant targets indicates that integrating optimal control formulation into RL has the ability to replicate well-known human arm movement properties and our results show strong matches to the experimental velocity profile. It can be observed in experimental data that human subjects have tendency to reach the target earlier than normalized time window and stabilize the hand with remaining time. Similar observation can be seen in simulation results, however with negligible less stability. In this experiment, we also showed that the inverse bell-shaped velocity profile for reaching tasks is captured in our learning and optimization framework.

**Fig. 5:**
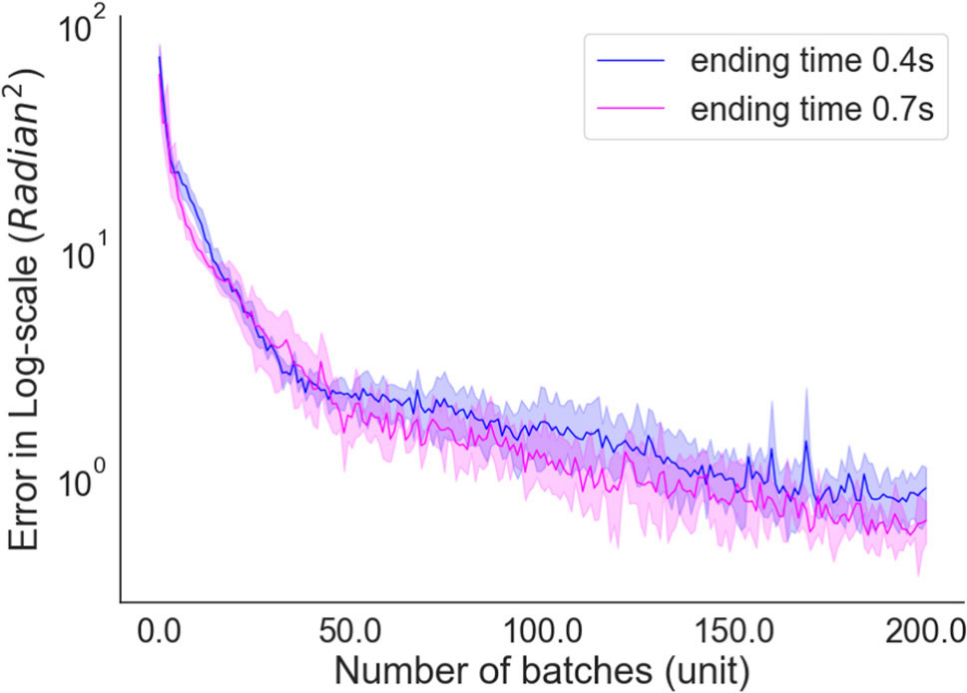
Learning curve of precise timing control experiment. Two experimental setting is provided, blue line with 0.4 seconds ending time, as well as magenta line with 0.7 seconds ending time. The scale of error is given in log-scale with respect to batch numbers. For each experiment, we performed 10 training and provided the mean (dark lines) and confidence interval (light shading). In both settings, learning curved converged to a stable solution where the acceptance rate is below 0.1

### Precise timing control

**Fig. 6:**
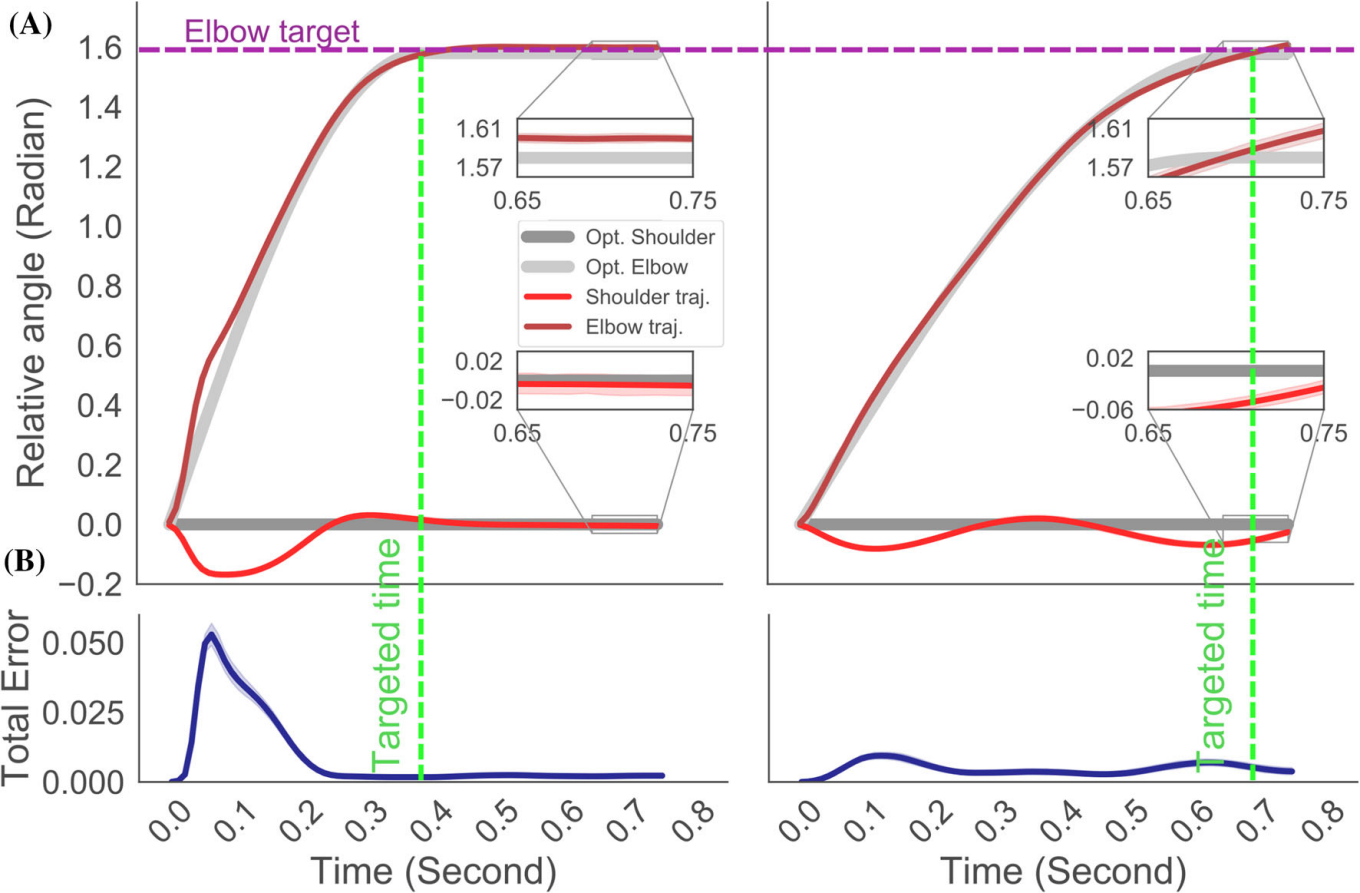
Precise timing control with 0.4 and 0.7s target time to reach the goal positions. A. The results are given as average of ten execution of the trained neural network for both experiments. The elbow target is indicated with a purple dashed line while targeted time is given with a green dashed line for both experimental settings. For each experiment, the difference between the desired trajectory and simulation results is given in the inner figures, the range of difference is negligible. Both optimal shoulder and elbow trajectories are also provided with grey lines to compare the results visually. B. The error of the difference between the desired and actual trajectories in elbow and shoulder joint is summed and averaged for all execution of the trained neural network. Due to higher speed at the beginning of the experiment in 0.4s target time, the shoulder joint shows higher variance of movement which is visible in the total error

The next experimental setting was inspired by a classical optimal control problem which requires an end point control within a given time. The goal is defined as to reach the same target position in space albeit with different timing. We performed four different experiments with identical goal position with a different final time (see Eq. 3 the term “n”). We only provide two experiments; however, remaining two other experiments can be found in Supplementary material, Sect. 4. In all experiments, the tip of the hand of the arm is required to reach $\pi /2$. In this experimental setting, the upper arm is needed to stay stable while only the forearm was allowed to move. The complexity of this problem arises due to the momentum compensation at the upper arm created by the lower arm. This constraint is integrated into the optimal control formulation as a path constraint (see Eq. 3 the Lagrangian term”) for the upper arm. In Fig. 5, the evolution of the error function is given. Since the definition of the reward function in our formulation is the exponential difference between the optimum trajectory and the current trajectory, reward function is interpreted as error function and the objective is to minimize this difference. The results of two different settings with different final time are averaged across 10 experiments for each settings with different random seeds. As the learning curve of both setting indicates, the best results are achieved after approximately 150 batch iterations where each batch size is 64. All the parameters of this experiment can be found in Supplementary material, Sect. 3. The results with the other two experiments that are given in supplementary also show that the learning curve of this experiment is final-time invariant. Regardless of the ending-time goal (see Eq. 3 the term “n”), the Deep RL agent has the capability of converging to a stable solution within similar number of batches with low variance which is defined empirically by looking at the difference between two consecutive batches and stopping the learning process when the change of error is less than a certain value that is defined empirically as well. This variance can still be reduced to certain amount by better optimizing the hyper-parameters, running the learning experiment longer than 200 batches as well as deploying it to a high-performance computing clusters that are suitable for Deep RL simulations.

The solutions that we obtained with numerical optimal control are given in Fig. 6A with grey lines as well as the goal of the elbow joint with a purple dashed line along with time constraints in green dashed line. The target for the shoulder joint is zero for both cases. The generated movement of the musculoskeletal arm is also given in Fig. 6A where 10 testing results of the movement are averaged for both elbow and shoulder joints. Although the optimal solution has the ability of stabilization in the upper arm perfectly as it can be seen in Fig. 6A, the musculoskelatal movement has slight disturbance in the upper arm due to limitation of the muscles in 2D control setting.

The time dependent evolution of the joint trajectories and corresponding error can also be found in Fig. 6B and, where we provide all the outcome of the two different experimental settings. The main difference between each of these different joint trajectories is the muscle activity profile found by the Deep RL . Since there exists a path constraint (see Eq. 3 the Lagrangian term) that defines the initial condition and the final condition on state values with given final time constraints, the position and velocity profiles are obtained accordingly. As it can be followed, the solution of the numerical optimal control forces system to start with high acceleration of the elbow joint in the first experiment to satisfy the end-timing condition of 0.4s while maintaining the position of both joints after the target time, whereas the elbow trajectory reaches the target position with a time-lag due to the target time of 0.7s. In both experimental settings, the shoulder joint oscillates around the target position of 0 radian, however with negligible variance of 0.2 and 0.1 radians, respectively. The stabilization of the shoulder joint requires us to consider remaining muscles of the shoulder joint in 3D joint control that we leave it as a future research.

In all experiments, the movement has been finalized at reaching the goal state with a low divergence from the target positions, therefore we also claim to solve the stabilization of the arm at the range of desired end point. The inner figures in Fig. 6A shows the difference between target trajectories and observed ones at the end of the simulation. It can be seen that the error between target and observation has the range of 0.04 radian for all experiments of all joints considered. To assess the robustness of the solutions, we performed 48 simulations of the same experiment. The simulations that has error below 0.1 have considered successful experiments. The total error of the above-mentioned experiment is given in Fig. 6B where the total error is less than 0.1 for both experiment. 44 simulations out of 48 have been labelled as successful experiments in which the Deep RL agent learnt to perform the desired movement in the musculoskeletal arm model.

**Fig. 7:**
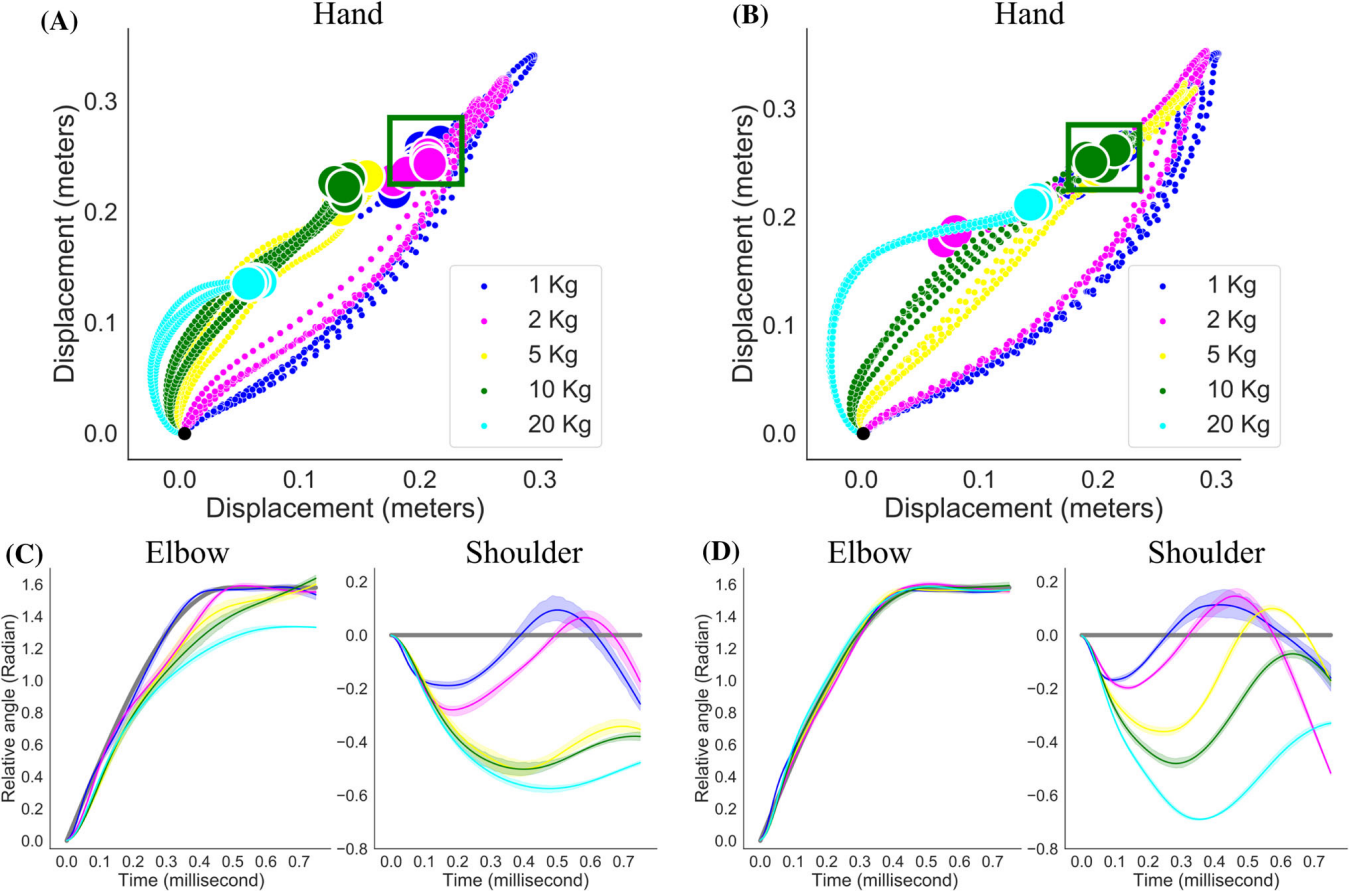
Weight lifting experiment with 1, 2, 5, 10 and 20 kilos. The results of two different parameter settings of the reward function in Deep RL. **A** Displacement of the hand with respect to initial position. Colour codes indicate different weights. The goal position is indicated with a green square. The musculoskeletal arm manages to lift 1 and 2 kilos only. Each experiment is repeated 5 times and each of them provided. **B** Similar to A but the parameters of the reward function prioritize the elbow trajectory. 1, 2 and 5 kilos are lifted; however, there exists overshooting in the experiment with 2 kilos. **C** Trajectories of the elbow and shoulder joints with identical parameters of the reward function $w_{q,e} = {\dot{w}}_{q,e} = \ddot{w}_{q,e} = 1; w_{q,s} = {\dot{w}}_{q,s} = \ddot{w}_{q,s} = 1$. **D** Similar to C except elbow joint has higher coefficients in the reward function, $w_{q,e} = {\dot{w}}_{q,e} = \ddot{w}_{q,e} = 1; w_{q,s} = {\dot{w}}_{q,s} = \ddot{w}_{q,s} = 0.2$. The elbow joint control shows increased performance

### Weight lifting

In all the experiments reported so far, the control problem solely depends on the joints’ movement without external perturbation and would therefore integrated into musculoskeletal control such that the only requirement is to deploy necessary muscle activation to achieve smooth movement trajectories. Here, we investigated the capability of the proposed learning and optimization framework in case of the existence of an additional disturbance from the environment, hence the requirement is to lifting an object with varying weights in the hand of the musculoskeletal arm. Here, we attached an object to the hand of the musculoskeletal arm using weld joints to create a joint movement between the arm and the object. The utility of coordinated muscle activities for lifting objects while performing an identical movement trajectory becomes more intuitive since it shows us the recruitment of the correlated muscle activities with the amount of weight on the hand of the musculoskeletal arm. The goal of this setting is to show the ability of the learning and optimization framework to gradually recruit necessary muscles to accomplish given lifting tasks. In this experiment, we gradually increased the weights from 1 kilo up to 20 kilos to adjust the neural controller to incrementally more challenging settings. For each lifting scenarios, we tested the learned neural controller 5 times to analyse the robustness of the learned model. We also investigated the effects of the parameters of the reward function on the performance of the neural controller (see Eq. 6).

In the first settings, we keep all the parameters of the reward function identical and set them to 1, and results are given in Fig. 7A and C. Since there is no difference between the weight of shoulder and elbow movements, the neural controller optimizes both trajectories without giving any priority to any of the joints. Up to 5 kilos, the musculoskeletal arm managed to successfully lift the objects to the desired target position and keep the hand at the target. Once we adjust the reward function in favour to the elbow joint movement, $w_{q,e} = {\dot{w}}_{q,e} = \ddot{w}_{q,e} = 1; w_{q,s} = {\dot{w}}_{q,s} = \ddot{w}_{q,s} = 0.2$, the neural controller achieved perfect control on the elbow joint while performing a poor control on the shoulder joint (see Fig. 7D). Due to the parameters of the muscles, there is a cut-off weight that the musculoskeletal arm can carry which is approximately 5 kilos as it can be seen in Fig. 7A and B. The individual muscle activity of the all 14 muscles for both experiment is given in the Supplementary material, Sect. 4. We observe that in this experimental settings there exists highly correlated activity among flexor muscles both in shoulder and elbow joints, mainly between BICLong, ANC, TRIMed, TRILat and PL with varying time lag. At the beginning of the experiment, upper arm extensors start highly active and diminishes at the end which indicates the recruitment of these muscles to stabilize the shoulder joint as the goal requires. As well, upper arm flexors have ranging activity to contribute the stabilization of the shoulder joint; specifically, BRD muscle provides baseline activity throughout the trajectory in all lifting settings. We also observed that only PL and FCR forearm flexors are recruited in the case of successful lifting experiments and stay silent for the remaining lifting experiments.

**Fig. 8:**
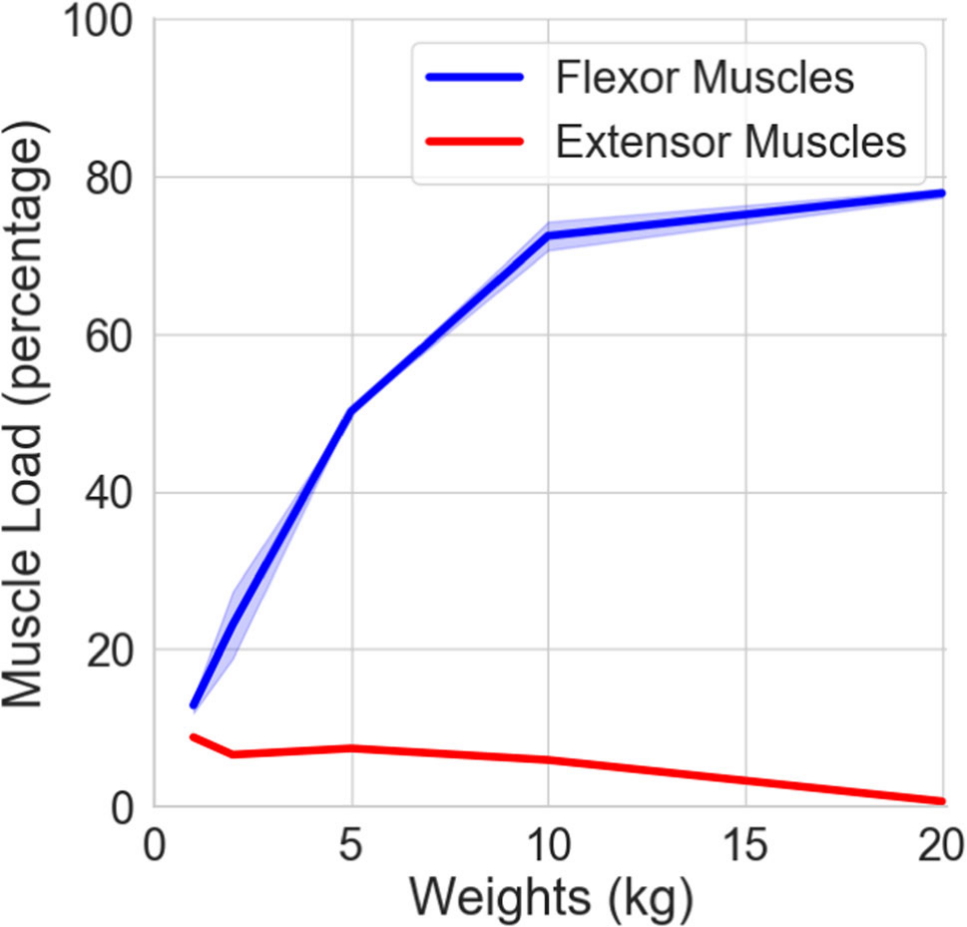
Muscle load with respect to weights. The recruitment of the flexor and extensor muscles is given in blue and red, respectively. The flexor muscle recruitment is approximately linear to the weight load up to 10 kilos; then it saturates around $\%80$. Extensor muscles are recruited for low weights; then it gradually diminishes at around 20 kilos

We also investigated the recruitment strategy of the neural controller on flexor and extensor muscles during the lifting experiments. We recorded all 14 muscle activities during one episode of the movement and we repeated the recordings 5 times for each lifting experiments starting from 1 kilos up to 20 kilos. We then put a threshold value of $\%80$ out of maximum value of muscle activation; then we average the total amount of muscle activation above this threshold in all flexor and extensor muscles, respectively. By dividing the total recruitment of muscle activities to maximum possible muscle activation during one episode, we average the muscle load with respect to varying weights of the objects. We observed that increasing the weight of the object forces neural controller to recruit more maximally activated muscles and also adjust the recruitment strategy, similar to the observations in human subjects of experimental studies (Lawrence and DeLuca 1983). It can be seen in Fig. 8 that recruitment of the flexor muscles shows approximately linear relationship with the weight of the objects that musculoskeletal arm is carrying up to 10 kilos; then it saturates around $\%80$ of muscle load. We also observed that extensor muscles are only partially recruited but this recruitment diminishes by increasing the weights of the object. This recruitment strategy of the neural controller indicates that extensor muscles are activated in order to stabilize the movement and also adjust the deceleration of the arm; however, it is omitted once the movement requires higher recruitment of the flexor muscles to prioritize achieving the target point instead of stabilization of the arm movement. As it can be followed in Fig. 8C and 8D, the optimizer first prioritizes the optimization of the elbow joint while the shoulder joint optimization is less prioritized. We observe such difference between these two joints due to the fact that elbow and shoulder joints are constituting a coupled mechanical system with limited muscle activity. At the stage where the elbow is optimized, it is no longer possible to bring the shoulder joint to an optimized level due to the limitation of the existing muscle forces.

### Obstacle avoidance

Up until this point, we focused on tasks that require an arm movement to a single target in state space and discussed the performance and shortcomings of the neural controller. One of the most striking abilities of optimal control formulation is that the objective of the task can be enlarged to sequential decision problems such that a consequential target positions can be tackled. Here, we present an experiment to study the ability of the optimization and learning framework on sequential target achieving problems where the goal position was blocked by an obstacle. During the execution of the movement no such time limitation was assigned although it can be incorporated to the formulation with additional constraint.

**Fig. 9:**
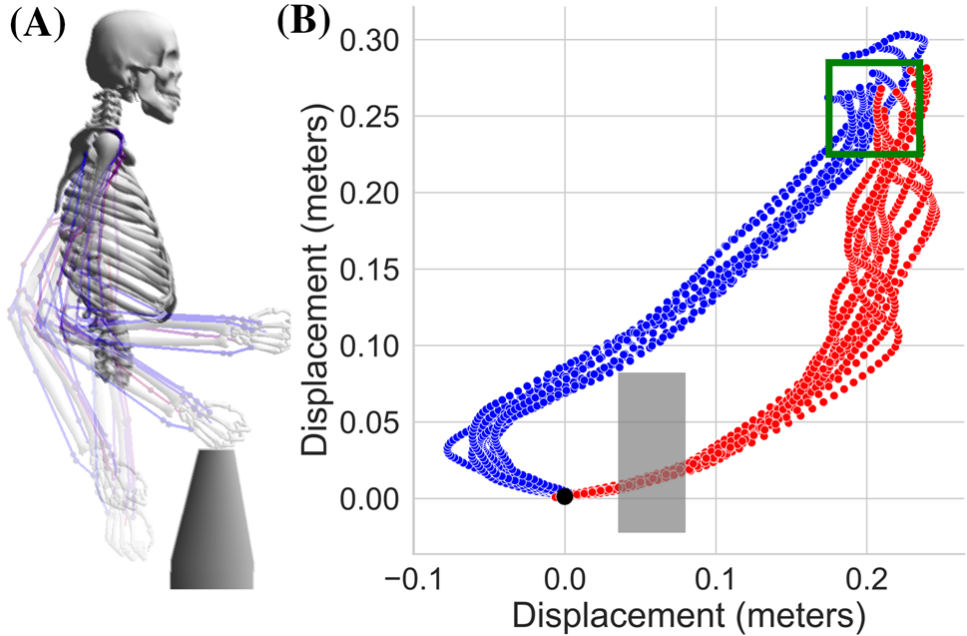
Obstacle avoidance task. **A** The schematic representation of the musculoskeletal arm movement. Only 4 snapshots of the movement are given for the sake of visual clarity. The obstacle is given in grey block. **B** The simulation results of the hand displacement in case of obstacle avoidance in blue. Red trajectories represents the same task without an obstacle. Each task is executed 10 times

Figure 9 shows the simulation results of movement control in case of an obstacle as well as hand trajectories without the existence of an obstacle for compare reasons.

To incorporate the obstacle avoidance in the formulation of the MPC, we added an additional set point that introduce the position of the obstacle in state space, $x^{obs}$, as an inequality constraint such that difference between the joint positions and border of the obstacle must be greater; then a constant value hence the objective function is written as below;

8 $$\begin{aligned}&\underset{x,u}{min}\sum _{k=0}^{n-1}\Big ( (x-x^{*})^2 + 10*(x-x^{obs})^2\nonumber \\&+ \varDelta u_{k}^T R \varDelta u_{k} \Big ) + (x-x^{*})^2 \end{aligned}$$

where the target position of the tip of hand, $x^{*}$, alternates given a switch set point, $t_{s}$;

9 $$\begin{aligned} \left\{ \begin{matrix} x^{*} = \pi /8 \quad \text {if} \quad t<t_{s}\\ x^{*} = \pi /2 \quad \text {if} \quad t\ge t_{s} \end{matrix}\right. \end{aligned}$$

In addition, the challenge for the neural controller to integrate the solution of the MPC is to reverse the extensor and flexor activities of the muscles in order to obtain swinging movement during the episode. As the simulation results indicate, we obtained hand movements that satisfies the above inequality constraints as well as a minimum value for the objective function. The hand movement that we obtained follows a trajectory which avoids the obstacle while bringing the tip of the hand to the indicated goal position.

## Discussion

In this work, we focused on a 2D musculoskeletal human arm model with 14 extensor and flexor antagonistic muscles. We presented a novel approach to tackle the control of the musculoskeletal systems based on model predictive control and deep reinforcement learning. We used the explicit formulation of MPC to obtain efficient trajectory optimization of skeletal systems using direct collocation as the nonlinear programming solution of the MPC. We then used the solution of the optimum joint trajectories in the simulation of centre-out reaching task, precise timing control, weight lifting and obstacle avoidance problems with muscle recruitment of a deep neural network that has been trained by PPO. We demonstrate the efficacy of our solution by analysing the capabilities of the neural network on trajectory tracking problems.

From robotics point of view, there are two main methodologies to identify the controller of the musculoskeletal joints: a model-free metaheuristic optimization or mathematical optimization methods that requires the knowledge of system dynamics. The advantage of model-free metaheuristic optimization methods is that one does not need to know the details of the musculoskeletal system’s kinematics and dynamics to solve the control problem. Thanks to the recent improvements in computational power, one can solve a model-free optimization problem on a computer cluster and achieve promising results in an acceptable time. There has been several successful studies to solve the musculoskeletal control problem with metaheuristic optimization methods and evolutionary algorithms (Coros et al. 2011; Geijtenbeek et al. 2013; Dura-Bernal et al. 2017). In these studies, it has been shown that a solution of the musculoskeletal control can be obtained with these algorithms. However, these studies only focus on the solution of the control problem without biological concerns. In addition, the solutions are restricted to the mechanical properties of the system, such that a change in the musculoskeletal system requires a training from the beginning since these methods do not incorporate the dynamics of the model into the optimization procedure. However, we focused on one of the motor control hypotheses, OCT, to address the biological relevance of the movement trajectories as well as the methodology of the OCT allowed us to incorporate the system dynamics of the musculoskeletal system in which we showed the motor control model has the ability to generalize the movement trajectories while satisfying the minimum energy consumption.

Our experiments show that reformulating the reward function of Deep RL provide a solution of mapping the optimal trajectories to the muscle control. Instead of using a global reward function that determines the desired goal position, the reward function is written as a minimization of the difference between a reference trajectory and the current state positions. As it can be seen in one of the recent implementation (Heess et al. 2017), the movements that have been found by Deep RL can only achieve forward movement while controlling the joints in an obscure way. However, by using the trajectories from optimal control as a reference signal for Deep RL, our implementation has allowed us to acquire optimum and human like behaviour of the musculoskeletal system. It can be said that OCT not only satisfies the optimality condition but also consider the dynamics of the system itself and therefore provides physically realistic reference trajectories. The other advantage of using OCT is that all feasible movement trajectories can be obtained with reformulating the objective function and the related state and path constraints. This enables us not only to obtain the goal position but also to control the joints in every single time step. As a result of this precise control, we have managed to solve the ill-posed problem of muscle control where the activation of multiple antagonistic muscles results in a one dimensional joint trajectory. We have also obtained the time coarse of activation for the individual stimulus for each muscle.

A similar idea has been gaining attention in the computer-animation where hand-crafted sequences of a musculoskeletal system are used as reference motions to be learned with Deep RL (Coros et al. 2011; Geijtenbeek et al. 2013; Peng and vande 2017). The caveat of this approach is that reference motions need to be created for all frames in a sequence. In addition, contrary to the solutions of Deep RL where the learned trajectory is one of many possible trajectories, we have shown that our framework can approximate a desired trajectory. We showed that the error range of the learned trajectories is approximately around 0.05 radian which indicates the ability of the controller to track the desired trajectory.

The proposed learning and optimization framework for muscle control can also be adapted to different musculoskeletal control problems, by changing, for example, the morphological details of a limb model and adapting the system dynamics accordingly. It is therefore also possible to study different animals, since the framework only requires to write equations of motions and its physiological constraints for the desired animal model. Therefore, the results for the human musculoskeletal system can be modified to study not only human models but also another mammalian musculoskeletal control problem. Apart from the fact that our approach depends on an abstract dynamical model of a musculoskeletal system, more comprehensive models of muscle path wrapping, more accurate models for proprioception signals and excluded sub-units of the motor control could be integrated into our framework to further improve the quality of the solution.

## Supplementary Information

Below is the link to the electronic supplementary material.

Supplementary file 1 (pdf 4380 KB)
