# Supplementary material for: Optimum trajectory learning in musculoskeletal systems with model predictive control and deep reinforcement learning
Source: Biol Cybern. 2022 Aug 11;116(5-6):711–26. doi: 10.1007/s00422-022-00940-x (PMC9691497; doi:10.1007/s00422-022-00940-x)
Supplement: Supplementary file 1 — (pdf 4380 KB) [file 422_2022_940_MOESM1_ESM.pdf]

Supplementary Material: Optimum trajectory learning in musculoskeletal systems with Model Predictive Control and Deep Reinforcement Learning

Berat Denizdurduran, Henry Markram, Marc-Oliver Gewaltig

1 Model Details

1.1 Multi-body Dynamics of the Musculoskeletal System

The equations of motion that describe the multi-body dynamics of the musculoskeletal system is given as follows:

$$M(q)\ddot{q} + C(q, \dot{q})\dot{q} + G(q) - \tau = 0 \tag{1}$$

where $q, \dot{q}, \ddot{q}$ denotes angles, velocities and accelerations of the joints, $M(q)$ as the mass matrix, $C(q, \dot{q})$ describes the Coriolis forces along with $G(q)$ gravity force and as well as the joint torques, τ that are generated by muscle contractions. The transformation of muscle forces to the joint torques are executed within the forward dynamics simulation engine of the OpenSim [1].

1.2 Muscle Dynamics

The musculoskeletal arm model is controlled by 14 extensor and flexor muscle-tendon units, abbreviation of these muscles and also the nomenclature of the paper is given in (Table 1).

Incorporating the properties of muscle dynamics into mathematical models pave the way for musculoskeletal simulations where different hypothesis of the underlying neural motor control can be examined. There exists a large number different muscle models, that differ in the level of description, their complexity and behavior being modelled. The difference of most models are either focus on how microscopic processes at the level of the tissue generate function or generic functional models that describe the input-output relationship between muscle activation and force generation [2]. The microscopic level of modeling approach ofmuscles takes into account the cross-bridge dynamics and the sliding activity of the filaments, e.g. Huxley muscle [3] whereas the generic macroscopic models focus on more high level properties of the muscles such as the force-length and the force- velocity relationships e.g. Hill-type muscles [4, 5, 6, 7].

Table 1: Nomenclature of the paper

Abbreviation	Name
CNS	central nervous system
RL	reinforcement learning
Deep RL	deep reinforcement learning
OCT	optimal control theory
NN	neural networks
MTU	muscle-tendon units
MPC	model predictive control
MDP	markov decision process
DOF	degrees-of-freedom
PPO	proximal policy optimization
TRILong	triceps Long
TRIMed	triceps Medial
TRILat	triceps Lateral
BICLong	biceps Long
BICShort	biceps Short
BRA	brachialis
ANC	anconeus
BRD	brachioradialis
ECRL	extensor carpi radialis longus
ECRB	extensor carpi radialis brevis
ECU	extensor carpi ulnaris
FCR	flexor carpi radialis
FCU	flexor carpi ulnaris
PL	palmaris longus

Since the microscopic properties ofthe muscle tissue are not relevant to biomechanical simulation studies, so-called Hill-type muscle models are widely used instead ofmicroscopic models. Sensory information can also be extracted from these models due to the fact that the length, velocity and force are calculated within these models.

Muscle model consists of several so-called MTUs. The MTU defines the dynamic properties of a muscle. An MTU has two parts: an elastic part (the tendon) and contractile part, (muscle unit). These two parts are serially connected. The muscle unit is again composed of two parts: a contractile part and an elastic element, but this time they are in parallel. The combined activity of those sub-units generates the force that causes the movement in the skeletal system. Therefore, muscle force is generated by these serial and parallel elements of the MTU. The contractile element $f^L(l^M)f^V(l^M)$ with parallel elasticity element $f^{PE}(l^M)$ composes the muscle unit which in turn generates the muscle force f^M. The parallel elasticity element becomes active in case of excessive stretches in the contractile element or shrinkage of the contractile

element beyond the acceptable range in order to prevent muscles tendon unit to be collapsed. In addition, serially connected tendon unit provide series elasticity $f^T(l^T)$ which in turn creates tendon force f^T and it takes part in the force-length and force-velocity profile of the muscle.

The activation of the muscle is driven by the stimulus of the motor neurons as a first order differential equation which takes into account the neural delay with parameter τ:

$$\dot{u} = \frac{a - u}{\tau} \tag{2}$$

The contraction of the muscle depends on the joint states of the musculoskeletal dynamics, which in turn depends on the states of the muscles. Therefore we are faced with a recurrent nonlinear dynamical system. According to the Hill-type muscle modelling, the force is cal- culated while considering the force-velocity, force-length and the active stimulation of the muscle as given below.

$$f^M = f_0^M(uf^l(l^M)f^v(v^M) + f^{PE}(l^M) + \beta v^M)\cos\alpha \tag{3}$$

where f_0^M is the peak force at length l_0^M. After the integration of the muscle and joint positions and velocities, the continuous time-series of the stimulation is translated into the force, generated by the muscle sub-units. Hence, the activation ofthe muscle by a stimulus generates the active tension in the muscle model with the evaluation of the active contractile element, a passive elastic element and the tendon dynamics.

The force-length relationship of the muscle is a result of the properties of sarcomeres which is the main cause ofthe active force generated by the muscles and it has bell- shaped curve and it creates passive force after a certain point of stretching. The equation that represents the force-length relationship of the muscle is given as follows:

$$f^l(l^M) = \exp\left(c\left|\frac{l^M - l^{opt}}{l^{opt}w}\right|^3\right) \tag{4}$$

where l^{opt} is the optimum length of the muscle and w is the parameter for the force-length relationship of the sarcomere.

It can be seen that the total force exerted by the muscle is the combination of the active and passive forces generated by the contractile element and the passive elastic element. Muscles have also dynamic properties such that there is a relationship between the shortening velocity of the contractions and the force generation which represents the actin-myosin cycle. It has the effect on the force generation during nonisometric contractions. Instead ofa bell-shape curve, force-velocity has an inverted sigmoidal property. One important aspect of the force generation during the nonisometric contraction is that the force is generated if the muscle is stretched more than the threshold length regardless of the situation of the muscle, whether it is activated or not. The force-velocity relationship of the muscle is given by $f^v(v^M)$ which represents the concen- tric contraction, represented as inverted sigmoid.

$$f^v(v^M) = \begin{cases} \frac{v^{max} - v^M}{v^{max} - Kv^M} \\ N + (N-1)\frac{v^{max} - v^M}{7.56Kv^M - v^{max}} \end{cases} \tag{5}$$

where v^{max} is the maximum possible velocity and with parameters N and K. The passive force-length $f^{PE}(l^M)$ curve is given by as follows.

$$f^v(v^M) = \begin{cases} k_1(\exp(k_2 l^M) - 1) \\ 0 \end{cases} \tag{6}$$

The new length of the muscle after one time interval $[t_n, t_{n+1}]$ depends on the contraction velocity ofthe muscle. In order to calculate the numerical new length, length-velocity equation is solved with trapezoidal rule as follows.

$$l^M(n+1) = l^M(n) + 1/2\Big(t_{n+1} - t_n\Big)\Big(v^M(n+1) + v^M(n)\Big) \tag{7}$$

and the velocity of the muscle is found by the inverse of the muscle force-velocity equation as follows.

$$f^v(v^M) = \begin{cases} v^{max}\frac{1-f^v}{1+Kf^v} \\ v^{max}\frac{f^v - 1}{7.56K(f^v-1)+1-N} \end{cases} \tag{8}$$

The muscle model are represented by multiple compartments in the simulation while the attachment sites are consistent with digitized muscle insertions and anatomical descriptions [8, 9]. The parameters of each muscle in this simulations are given in Table 2

2 Simulation Details

2.1 Model Predictive Control

The objective function of the skeletal control in Fig.6 for the precise timing control problem is given as follows:

$$\underset{x,u}{min} \sum_{k=0}^{n-1} \Big(1000 * x_{theta,0}^2 + (x_{theta,1} - \pi/2)^2\Big) + \Big(u_{force,1}\Big)^2 \tag{9}$$

2.2 Deep Reinforcement Learning

Derivation of the Deep Reinforcement Learning from Equation 5 in main manuscript can be written as follows [10]:

$$\nabla_\theta \underset{\tau\sim\pi_\theta}{\mathbb{E}}[r(\tau)] = \underset{\tau\sim\pi_\theta}{\mathbb{E}}[\nabla_\theta \log(\pi_\theta(\tau))r(\tau)] \tag{10}$$

The right-hand side of this equation is usually estimated instead of analytically solved by obtaining sample trajectories while executing the policy function as follows:

Table 2: Parameters of the muscles

Abbreviation	PCSA (cm^2)	Peak Force (N)	Optimal fiber length (cm)	Tendon slack length (cm)	Pennation (^{o})
TRILong	5.7	798.5	13.4	14.3	12
TRIMed	4.5	624.3	11.4	9.1	9
TRILat	4.5	624.3	11.4	9.8	9
BICLong	4.5	624.3	11.6	27.2	0
BICShort	3.1	435.6	13.2	19.2	0
BRA	7.1	987.3	8.6	5.4	0
ANC	2.5	283.2	2.7	1.8	0
BRD	1.9	276	17.3	13.3	0
ECRL	2.2	337.3	8.1	22.4	0
ECRB	2.2	252.5	5.9	22.2	9
ECU	2.1	192.9	6.2	22.8	4
FCR	1.6	407.9	6.3	24.4	3
FCU	2.9	479.8	5.1	26.5	12
PL	0.6	101	6.4	26.9	4

$\widehat{g}_\theta =$

$$\mathop{\mathbb{E}}_{\tau \sim \pi_\theta} \Big[\sum_{(s_t, a_t) \in \tau} \nabla_\theta \log(\pi_\theta(a_t|s_t)) \cdot (Q_{\pi_\theta}(s_t, a_t) - V_{\pi_\theta}(s_t)) \Big] \quad (11)$$

where a baseline function (here the natural choice is the value function) $V_{\pi_\theta}(s_t)$ is subtracted from the expected return $Q_{\pi_\theta}(s_t, a_t)$ in order to alleviate possibility of very large variance in the expectation [11]. It is also referred as advantage function:

$$\widehat{A}_{\pi_\theta}(s_t, a_t) = Q_{\pi_\theta}(s_t, a_t) - V_{\pi_\theta}(s_t) \quad (12)$$

Among several other possibilities (e.g. total reward of the trajectory, reward following action a_t, state-action value function), it is empirically shown that advantage function yields the lowest possible variance in the expectation. This function could be interpreted as forcing the gradients in a way that it is bounded to increase the probability of better than average decisions while decrease the probability of worse than average decisions. Given that expected return is the sum of discounted reward, one can rewrite the advantage function as follows:

$$\widehat{A}_{\pi_\theta}(s_t, a_t) = r_t + \gamma r_{t+1} + ... + \gamma^{T-t+1} r_{T-1} - V_{\pi_\theta}(s_t) \quad (13)$$

In case of choosing $\lambda = 1$ [12], one can come up with a more generalized version of the advantage function such that:

$$\widehat{A}_{\pi_\theta}(s_t, a_t) = \delta_t + (\gamma\lambda)\delta_{t+1} + ... + (\gamma\lambda)^{T-t+1}\delta_{T-1} \quad (14)$$

where $\delta_t = r_t + \gamma V_{\pi_\theta}((s_{t+1}) - V_{\pi_\theta}((s_t)$ is the temporal difference error. One way to simplify the maximization of expected return is to write down a surrogate optimization problem where focus is to maximization of the true reward [11]:

$$\mathop{max}_{\theta} \mathop{\mathbb{E}}_{(s_t, a_t) \sim \pi} \Big[\rho_t(\theta) \widehat{A}_{\pi_\theta}(s_t, a_t) \Big] \quad (15)$$

where $\rho_t(\theta) = \frac{\pi_\theta(a_t|s_t)}{\pi(a_t|s_t)}$ with current policy $\pi_\theta(a_t|s_t)$ and the policy before the update $\pi(a_t|s_t)$. There has been two main algorithms suggested to solve this maximization problem, Trust-region policy optimization (TRPO) [11] and ppo [12]. Although TRPO is easy to optimize, it is computationally costly due to the dependence of nonlinear conjugate gradient where multiple Hessian-vector products are required to be calculated. Instead, ppo instantly clip the objective function which means it doesn't require any conjugate gradient calculation by replacing Kullback-Leibler divergence constraint. We used ppo in our simulations in order to obtain muscle activities given desired joint trajectories. The maximization problem definition in the context of ppo can be given as follows:

$$\max_{\theta} \mathop{\mathbb{E}}_{(s_t,a_t)\sim\pi} \Big[min\Big(clip(\rho_t(\theta), 1-\epsilon, 1+\epsilon)\widehat{A}_{\pi_\theta}(s_t,a_t), \rho_t(\theta)\widehat{A}_{\pi_\theta}(s_t,a_t) \Big) \Big] \quad (16)$$

where ϵ provides the interval of the clip ratio. The first term is the conservative policy iteration which could lead to very large updates on policy optimization. To solve this problem, the second term is introduced to clip the probability ratio between the interval of $[1-\epsilon, 1+\epsilon]$.

We summarized all the hyperparameter settings for the PPO training in 2D human musculoskeleletal arm simulations in Table 3. We used a first-order gradient-based optimization of stochastic objective functions, called Adam [13] to estimate the gradients of the PPO.

Table 3: Hyperparameters of PPO training

Parameter	Value
initial weights w_0	$\mathcal{N}(\mu = 0, \sigma^2 = 0.1)$
critic loss coefficient	0.5
actor loss coefficient	1.0
entropy coefficient	0.001
hidden size	32
learning rate	$2e-4$
betas	(0.9 - 0.999)
eps	$1e-8$
weight decay	0.001
minibatch size	150
ppo epochs	150
ppo clip threshold	1.0
discount factor	0.95

3 Results

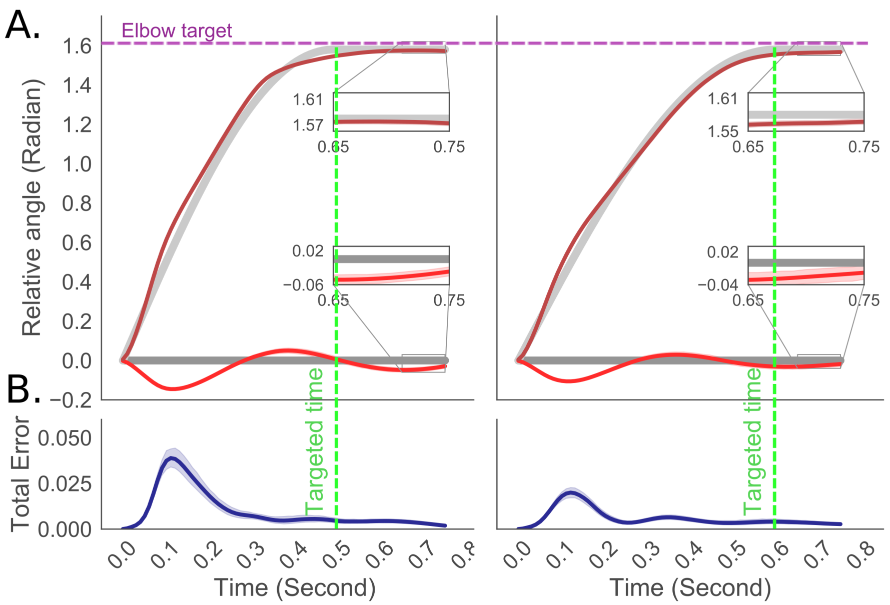

Figure 1: Precise timing control with 0.4s and 0.7s target time to reach the goal positions. A. The results of the remaining two experiments with similar problem settings of Figure 6 in the main paper. While the elbow target is indicated dashed purple line, the target of the shoulder joint is the x-axis since the shoulder joint is forced stay stable. For each experiment, the difference between the desired trajectory and simulation results are given in the inner figures where it can be seen that the resulting trajectories are close to optimal trajectories. B. The averaged error of both trajectories, elbow and shoulder can be seen in blue for both experimental settings. The magnitude of the initial error at the elbow joint is highly correlated with the targeted time of the goal position, more the elbow joint have time to reach the goal position, less the error at the beginning of the experiment.

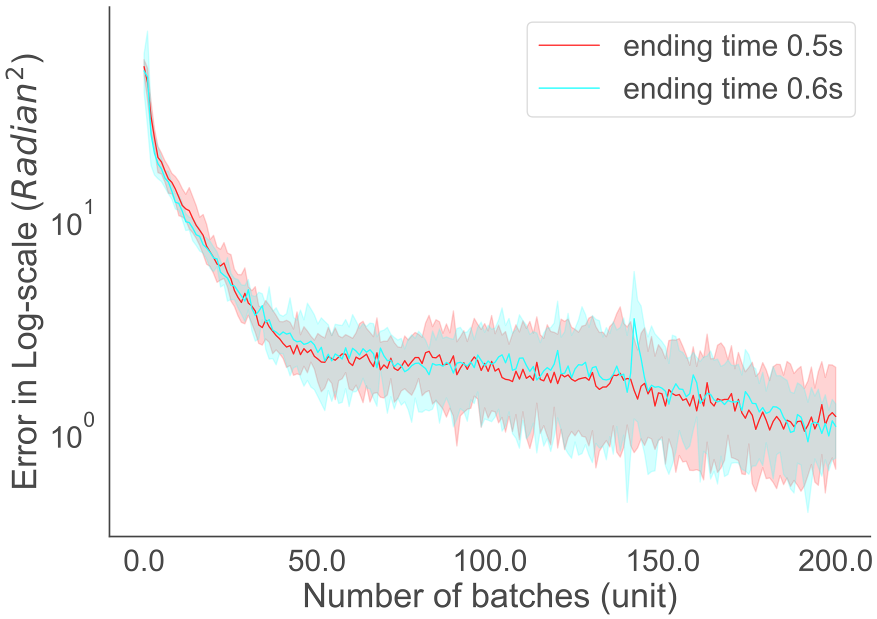

Figure 2: Learning curve of precise timing control experiment. Remaining two experimental setting is provided here, red line with 0.5 seconds ending time, as well as cyan line with 0.6 seconds ending time. The scale of error is given in log-scale with respect to batch numbers. For each experiment, we performed 10 training and provided the mean (dark lines) and confidence interval (light shading). Similar to the Figure 5 in the main paper, learning curve steadily converges to the acceptance rate of 0.1 after 150 batches.

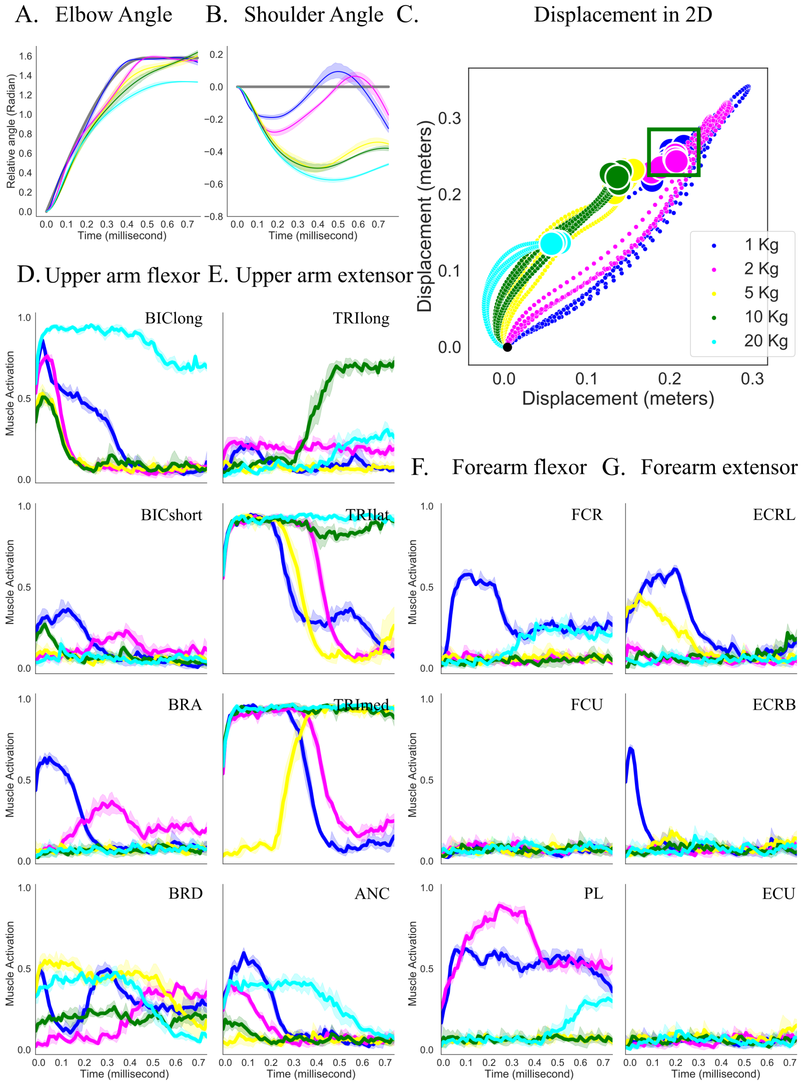

Figure 3: Weight lifting experiment with 1, 2, 5, 10 and 20 kilos where the parameters of the reward function is as follows: $w_{q,e} = \dot{w}_{q,e} = \ddot{w}_{q,e} = 1; w_{q,s} = \dot{w}_{q,s} = \ddot{w}_{q,s} = 1$ A. Trajectories of elbow angle for each weight lifting experiment B. Trajectories of shoulder angle for each weight lifting experiment C. Displacement of the hand tip point in 2D cartesian space for each weight lifting experiment. D. The recruitment of the upper arm flexor muscles, similar activity pattern observed except BIClong for weight of 20 kilos. E. The recruitment of the upper arm extensor muscles, almost maximum level of activity in TRIlong, TRIlat and TRImed for weights of 10 and 20 kilos. F. The recruitment of the forearm arm flexor muscles, FCR and Pl show activity only for the weights 1 and 2 kilos G. The recruitment of the forearm arm flexor muscles, ECRL and ECRB show initial activities at the beginning of the experiments for weight of 1 kilo.

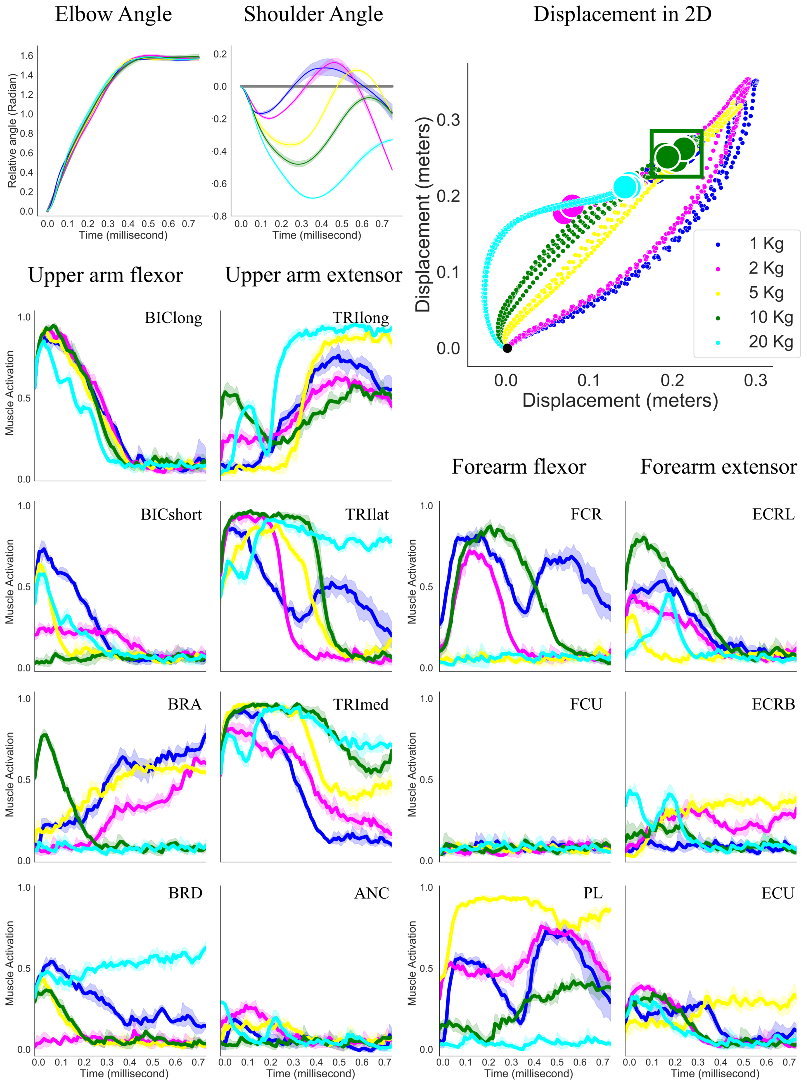

Figure 4: Weight lifting experiment with 1, 2, 5, 10 and 20 kilos where the parameters of the reward function is as follows: $w_{q,e} = \dot{w}_{q,e} = \ddot{w}_{q,e} = 1; w_{q,s} = \dot{w}_{q,s} = \ddot{w}_{q,s} = 0.2$. A. Trajectories of elbow angle for each weight lifting experiment B. Trajectories of shoulder angle for each weight lifting experiment C. Displacement of the hand tip point in 2D cartesian space for each weight lifting experiment. D. The recruitment of the upper arm flexor muscles, highly correlated activities for all muscles in BIClong. E. The recruitment of the upper arm extensor muscles, similar correlation among all the muscles except weight of 20 kilos F. The recruitment of the forearm arm flexor muscles, FCR and Pl are recruited at the beginning and at the end of the experiments G. The recruitment of the forearm arm flexor muscles, ECRL and ECU show highly correlated activities.

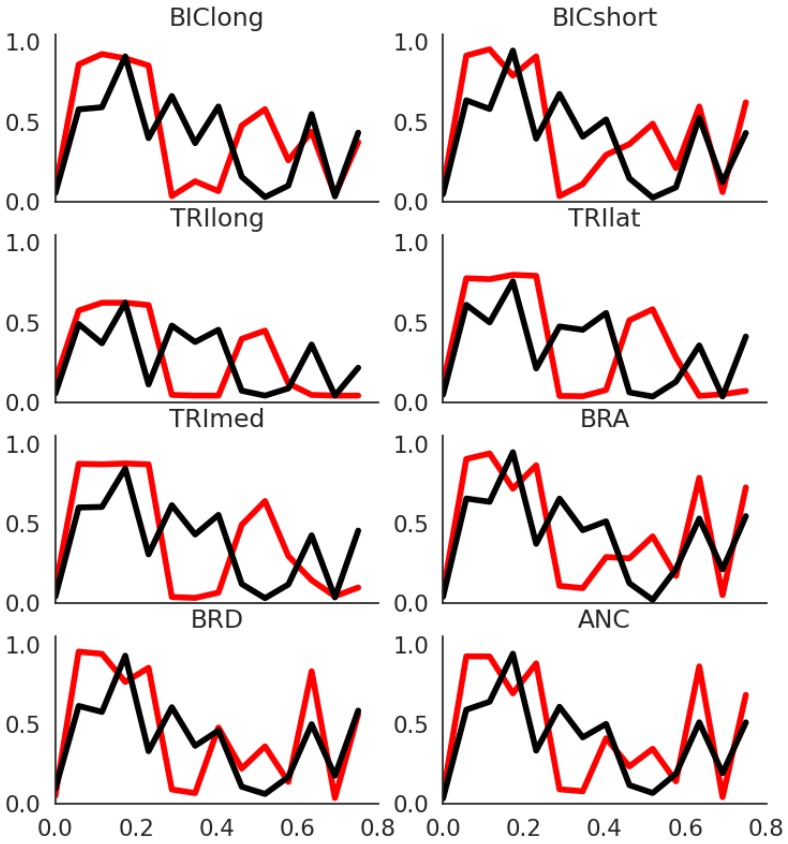

Figure 5: Comparison of flexor muscles activities for the experiments of precise timing control, see manuscript Figure 6. Red lines represents the muscle activity that is observed for the Experiment of reaching target within 0.4s, whereas black lines represents the muscle activity that is observed for the Experiment of reaching target within 0.7s. Since the target goal is identical but only timing differs, one can observe similarities of the activities in the muscles albeit more activity is observed for 0.4s since it requires faster movement.

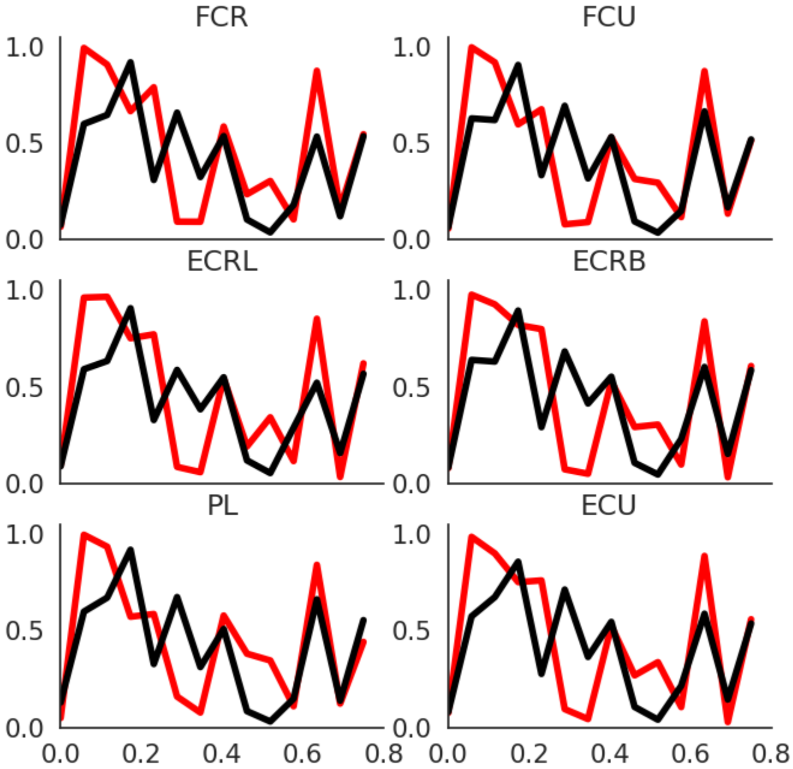

Figure 6: Comparison of extensor muscles activities for the experiments of precise timing control, see manuscript Figure 6. Red lines represents the muscle activity that is observed for the Experiment of reaching target within 0.4s, whereas black lines represents the muscle activity that is observed for the Experiment of reaching target within 0.7s. Similar to the activity of flexor muscles, there is also significant similarities in the extensor activities. One can also observe that all extensor muscles show high correlation between each other.

References

[1] A. Seth, J.L. Hicks, T.K. Uchida, A. Habib, C.L. Dembia, J.J. Dunne, C.F. Ong, M.S. DeMers, A. Rajagopal, M. Millard, et al., PLoS computational biology **14**(7), e1006223 (2018)

[2] F.E. Zajac, Critical reviews in biomedical engineering **17**(4), 359 (1989)

[3] A. Huxley, The Journal of physiology **243**(1), 1 (1974)

[4] H. Geyer, H. Herr, IEEE Transactions on neural systems and rehabilitation engineering **18**(3), 263 (2010)

[5] M. Millard, T. Uchida, A. Seth, S.L. Delp, Journal of biomechanical engineering **135**(2) (2013)

[6] D.G. Thelen, J. Biomech. Eng. **125**(1), 70 (2003)

[7] A.J. van den Bogert, K.G. Gerritsen, G.K. Cole, Journal of Electromyography and Kinesiology **8**(2), 119 (1998)

[8] W.M. Murray, T.S. Buchanan, S.L. Delp, Journal of biomechanics **35**(1), 19 (2002)

[9] K.L. Moore, A.F. Dalley, *Clinically oriented anatomy* (Wolters kluwer india Pvt Ltd, 2018)

[10] R.S. Sutton, A.G. Barto, *Reinforcement learning: An introduction* (MIT press, 2018)

[11] J. Schulman, P. Moritz, S. Levine, M. Jordan, P. Abbeel, arXiv preprint arXiv:1506.02438 (2015)

[12] J. Schulman, F. Wolski, P. Dhariwal, A. Radford, O. Klimov, arXiv preprint arXiv:1707.06347 (2017)

[13] D.P. Kingma, J. Ba, arXiv preprint arXiv:1412.6980 (2014)
